# Left-lateral thoracotomy for catheter ablation of scar-related ventricular tachycardia in patients with inaccessible pericardial access

**DOI:** 10.1007/s00392-020-01670-5

**Published:** 2020-05-26

**Authors:** Peng-Pai Zhang, Christian-Hendrik Heeger, Shibu Mathew, Thomas Fink, Bruno Reissmann, Christine Lemeš, Tilman Maurer, Francesco Santoro, YingHao Huang, Johannes Riedl, Michael Schmoeckel, Andreas Rillig, Andreas Metzner, Karl-Heinz Kuck, Feifan Ouyang

**Affiliations:** 1 Department of Cardiology, Asklepios Klinik St. Georg, Hamburg, Germany; 2 Department of Cardiology, Shanghai Xinhua Hospital Affiliated to Medical School of Shanghai Jiaotong University, Shanghai, China; 3 Medical Clinic II (Department of Cardiology, Angiology and Intensive Care Medicine), University Hospital Schleswig-Holstein, University Heart Center Lübeck, Lübeck, Germany; 4 Department of Cardiovascular Surgery, Asklepios Klinik St. Georg, Hamburg, Germany; 5 Fuwai Hospital/National Center of Cardiovascular Diseases, 167 North Lishi Road, Xicheng District, Beijing, 10037 China; 6 German Center for Cardiovascular Research (DZHK), Partner Site Hamburg/Kiel/Lübeck, Lübeck, Germany

**Keywords:** Ventricular tachycardia, Catheter ablation, Pericardium, Cardiac surgery

## Abstract

**Objectives:**

We aimed to describe the feasibility of a surgical left thoracotomy for catheter ablation of scar-related ventricular tachycardia (VT) in patients with inaccessible pericardial access.

**Background:**

Pericardial adhesion due to prior cardiac surgery or previous epicardial ablation procedures limits epicardial access in patients with drug-refractory VT originated from the epicardium.

**Methods:**

Six patients who underwent a surgical left lateral thoracotomy epicardial access for catheter ablation of VT after failed subxiphoid percutaneous epicardial access were reviewed. Patients’ baseline characteristics and procedural characteristics including epicardial access, mapping, and ablation were described. Epicardial access was successfully obtained in all patients by a surgical left lateral thoracotomy.

**Results:**

The reasons of pericardial adhesion were prior cardiac surgery (*n =* 3, 50%) and previous epicardial ablation procedures (*n =* 3, 50%). Epicardial mapping of the lateral and inferior left ventricle was acquired, and a total of 15 different VTs originated from those regions were abolished. Unless one patient with ST elevation myocardial infarction due to periprocedural occlusion of the posterior descending artery no further complications occurred. All patients were discharged 10.2 ± 4 days after the procedure. VT recurred in 1 patient (17%) and was controlled with oral amiodarone therapy during follow-up (median follow-up: 479 days).

**Conclusions:**

A surgical left lateral thoracotomy is feasible and safe for selected patients. This approach provides epicardial ablation in patients with VT located at the infero-lateral left ventricle and pericardial adhesions due to previous cardiac surgery or previous ablation procedures.

**Graphic abstract:**

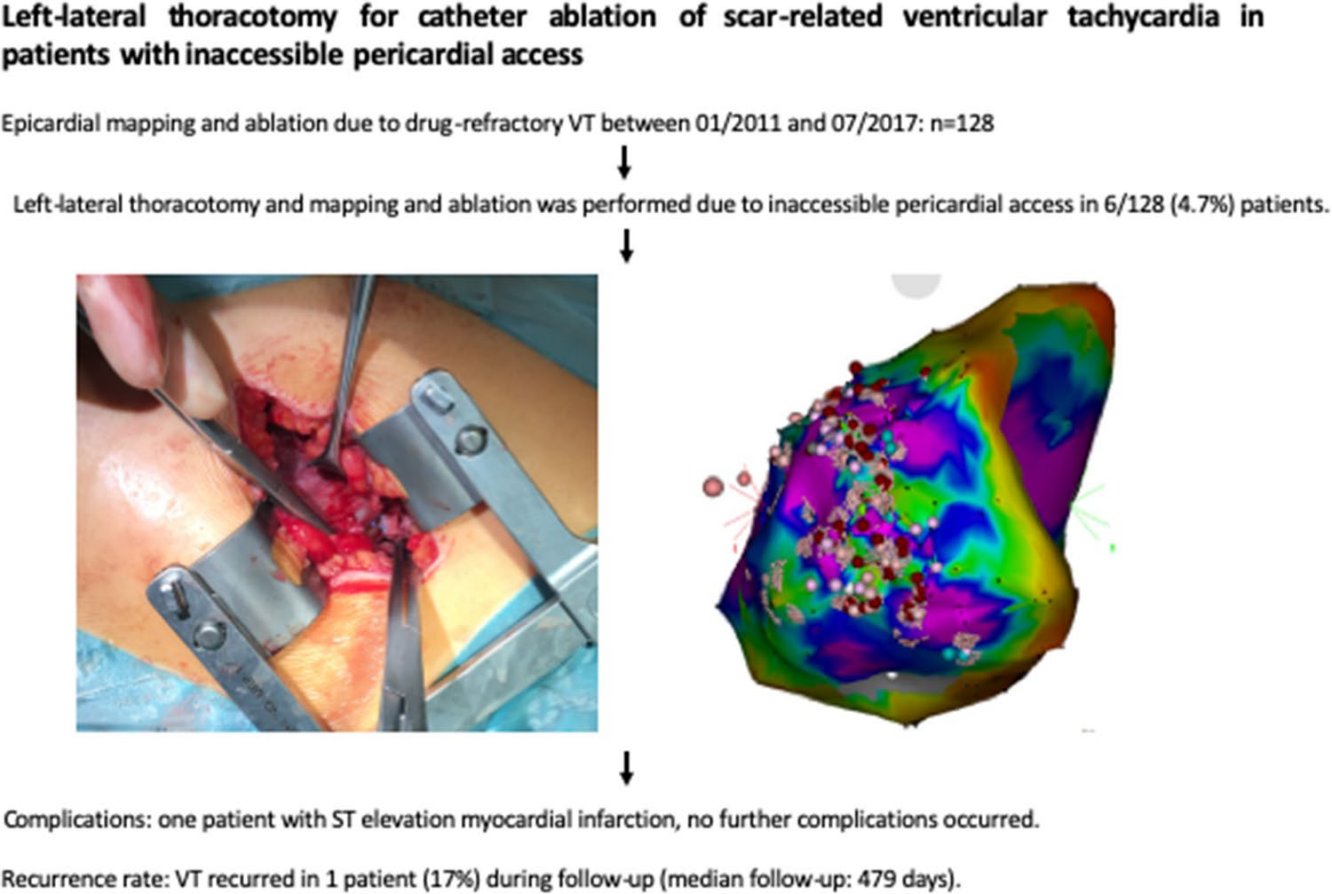

## Introduction

Percutaneous catheter ablation is a well-established therapy for recurrent ventricular tachycardia (VT) [1, 2]. Most VT can be abolished with radiofrequency (RF) catheter ablation by an endocardial approach [3–5]. However, epicardial mapping and ablation are necessary in 15–30% of patients when endocardial mapping techniques failed [6]. The percutaneous subxiphoid puncture technique is the most common approach to access the pericardium as first described by Sosa et al. [7]. However, in patients with pericardial adhesions, due to prior cardiac surgery or previous epicardial catheter ablation, the percutaneous subxiphoid puncture approach may be not successful or cause severe complications [8]. The purpose of this study was to describe a surgical left lateral thoracotomy to access the pericardial space for epicardial mapping and ablation in patients with scar-related VT located at the infero-lateral left ventricle (LV) and failed percutaneous epicardial access.

## Methods

### Patient characteristics

Epicardial mapping and ablation due to drug-refractory VT was performed in 128 patients at St. Georg hospital between January 2011 and July 2017. In 6/128 (4.7%) consecutive patients, a combined surgical epicardial access with mapping and ablation was performed. All other patients 122/128 (95.3%) received a subxiphoid epicardial puncture access. Surgical epicardial access was done in patients who met the following criteria: (1) recurrent VT with right bundle branch morphology resulted in electrical storms or multiple appropriate implantable cardioverter defibrillator (ICD) shocks refractory to antiarrhythmic drugs; (2) clinical VTs suggested an epicardial origin based on ECG criteria; (3) failed endocardial catheter ablation due to the absence of identifiable endocardial substrate or appropriately targeted sites; (4) attempted percutaneous epicardial ablation was unsuccessful by very experienced physicians because of the inability to enter the epicardial space by a subxiphoid puncture. Mapping and ablation were performed according to procedures approved by the human subject protection committee after patient consent was obtained. All patients gave written informed consent and all patient information was anonymized. The study was approved by the local ethic’s board (ethic’s approval number WF-44/17) and has been performed in accordance with the ethical standards given in the 1964 Declaration of Helsinki and its later amendments.

### Surgical access by left lateral thoracotomy

**Fig. 1:**
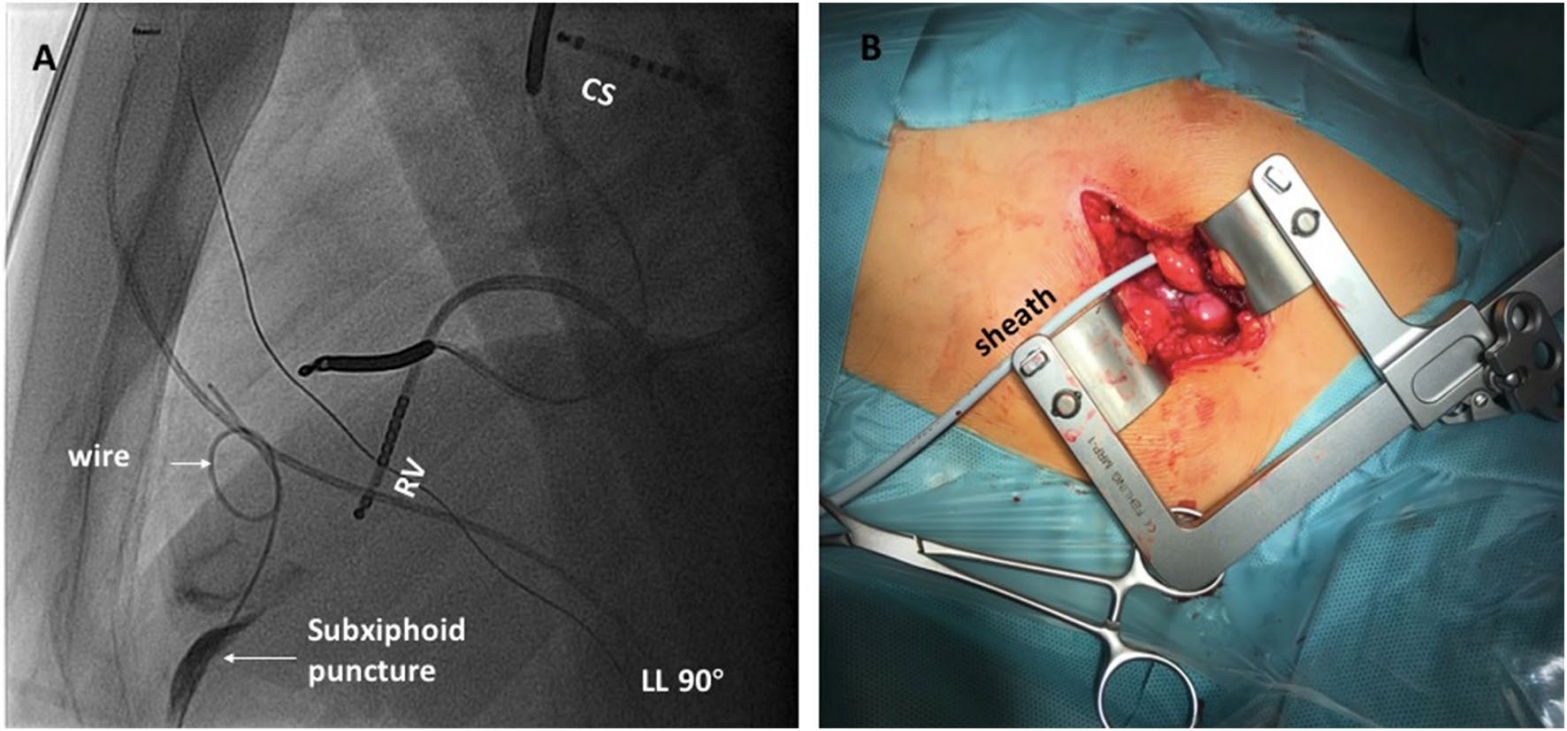
**a** Subxiphoid epicardial approach is shown, guide wire was confined due to the presence of pericardial adhesion. **b** Surgical left lateral thoracotomy is shown. A 5-cm incision was made at the left lateral fourth intercostal space, an 8F sheath was inserted into pericardial space, and a 7F mapping and ablation catheter was inserted into pericardial space via the sheath

No preprocedural imaging has been performed in all cases. All procedures were performed in a biplane electrophysiological lab. The patients underwent a surgical epicardial access as well as mapping and ablation under general anesthesia. Intubation was performed with a double-lumen endotracheal tube to allow for left lung deflation. Access was obtained by a surgical left lateral thoracotomy. A 5-cm long left lateral incision was made at the fourth intercostal space and extended through the subcutaneous tissue and fascia. The intercostal space was exposed and expanded for pericardial access. The pericardium was then exposed and dissected carefully. If necessary, blunt dissection of adhesions was performed to reach the area of interest. After the adhesions were separated, an 8F sheath was inserted into the pericardial cavity, and a 7F mapping and ablation catheter (3.5-mm tip, NaviStar, Biosense-Webster Inc., Diamond Bar, CA, USA) was inserted into the pericardial space through the sheath (Fig. 1). In every case, single-shot Cefazolin was prescribed. Pericardial drain and pleural cavity chest tubes were inserted when surgical closure was performed at the end of the procedure. The drainage tubes were removed on the next day if there were no bleeding events.

### Mapping and ablation

A multipolar diagnostic catheter was positioned at the RV apex and an electrophysiological study was conducted. Programmed stimulation was performed with up to triple ventricular extra stimuli at drive cycle lengths 510 and 400 ms, with 10 ms decrements down to 220 ms or refractoriness. An epicardial substrate voltage map was obtained during sinus rhythm using the CARTO 3 system (Biosense Webster) in all patients. Peak-to-peak bipolar voltage during sinus rhythm was defined as follows: normal tissue > 1.5 mV, dense scar < 0.5 mV, and border zone tissue 0.5–1.5 mV. [9] In patients in whom concomitant endocardial mapping was performed, access into the LV was obtained via a trans-septal and retrograde aortic approach.

After voltage mapping, VT induction was attempted with programmed stimulation. If induced VTs were hemodynamically unstable, termination was performed with burst pacing or external electrical cardioversion. The induced VT was defined as clinical VT if the 12-lead ECG morphology and cycle length (within 20 ms) matched the documented spontaneous VT.

For ablation of hemodynamically stable VTs, entrainment maneuvers were performed to identify the critical isthmus [10]; in case of unstable VTs, pacing at areas of late or fractionated potentials was used to identify the VT exit [11, 12]. Extensive ablation at the area with late or fractionated potentials was performed to eliminate these abnormal potentials. Irrigated-tip catheters (3.5-mm tip, ThermoCool; Biosense Webster) were used for epicardial RF-based ablation. Catheter irrigation during epicardial mapping was set at 2 ml/min. During epicardial ablation, power ranged from 30 to 45 W, with irrigation of 17 to 25 ml/min. Intrapericardial fluid was drained by aspiration from the surgical access periodically during the procedure. A coronary angiography was performed prior to epicardial ablation to assess proximity to coronary arteries if the target area was presumed to be close to the coronary artery main branches. At the end of the procedure, the initial stimulation protocol was repeated. Bipolar ablation was performed in case of epicardial and endocardial ablation failure and was performed by a bipolar ablation system (Stockert, Biosense Webster, Diamond Bar, CA). The first catheter was placed at the endocardium and the second catheter was placed at the corresponding site of the epicardium. The second ablation catheter was connected as the return electrode to the RF generator instead of to a dispersive patch.

Ablation was initiated endocardially with a power of 30 W, and then titrated up to a maximum of 45 W over 60–120 s for an impedance drop of up to 40Ω. Ablation was terminated or the power was titrated down for a sudden rise in temperature or a rapid fall in impedance. During ablation, power ranged from 30 to 45 W, with irrigation of 17–25 ml/min. The acute end-point of the procedure was non-inducibility of the clinical VT or slow VTs at programmed electrical stimulation.

### Follow-up

All patients regularly visited our outpatient clinic or the referred physician. ICD interrogation records were routinely used to determine whether the patients had recurrence of VT.

## Results

### Patient characteristics

Patient baseline characteristics are presented in Table 1. The six patients consisted of four men and two women, with a mean age of 55 ± 16 years (32–76 years). The mean LV ejection fraction (LVEF) was 40.7 ± 7.6% (20–55%). The underlying disease was idiopathic dilated cardiomyopathy (DCM) in four and ischemic cardiomyopathy (ICM) in two patients. All patients were ICD carriers and presented with recurrent VT or multiple adequate ICD shocks. In those patients, previous endocardial ablation was performed with one attempt in two patients (#3 and #6), with two attempts in patients #4 and #5, with three attempts in patient #1 and with nine attempts in patient #2. Previous epicardial mapping and ablation was performed in three patients (#1, #3, and #6). Cardiac surgery was previously performed in three patients with coronary artery bypass graft (CABG) surgery in patient #5 and with mitral valve reconstruction in patients #2 and #4 (Table 2).

**Table 1:** Baseline patient characteristics

Patients	Age(years)/gender	Etiology	LVEF (%)	Reason for ablation	Previous ablation (endo/epi)	Adhesions due to	ICD	Antiarrhythmic drugs
1	51/F	DCM	45	sVT	3/1	Epi ablation	Y	Sota, Flec
2	45/F	DCM	50	sVT	9/0	MV rep	Y	BB
3	32/M	DCM	55	sVT	1/0	Epi mapping	Y	BB
4	76/M	ICM	20	VT-Storm	1/0	MV rep	Y	Amio, BB
5	66/M	ICM	39	sVT	2/0	CABG	Y	Amio
6	60/M	DCM	35	sVT	2/2	Epi ablation	Y	Flec

*F* female, *M* male, *DCM* dilated cardiomyopathy, *ICM* Ischemic cardiomyopathy, *LVEF* left ventricle ejection fraction, *sVT* sustained ventricular tachycardia, *Epi* epicardial, *MV rep* mitral valve reconstruction, *Amio* amiodarone, *BB* beta blockers, *Sota* Sotalol, *Flec* Flecainide

**Table 2:** Characteristics of procedure

Patients	No. of VTs	Scar location, endo/epi	Complete epicardial mapping	Ablation endo/epi	Ablation approach	Procedural duration (min)	Acute result	Recurrence (yes/no)/follow-up (days)
1	6	Septal inferior LV and RV /posterobasal inferior and lateral LV	yes	+ / + , bipolar	Substrate + pacemap	230	No VT	(Yes)/1277
2	1	Inferior lateral basal LV/inferior lateral basal LV	no	+ / + , bipolar	Substrate + pacemap	325	No clinical VT, 1 VF inducible	(No)/1104
3	2	No/inferior lateral LV	yes	–/ + , unipolar	Substrate + pacemap	210	No VT	(No)/286
4	1	No/lateral and inferior LV	no	−/ + , unipoar	Substrate + pacemap	325	No VT	(No)/30
5	2	Lateral LV/superior lateral LV	no	+ / + , unipolar	Substrate + pacemap	200	No VT	(No)/120
6	2	No/posterolateral LV	no	−/ + , unipolar	Substrate + pacemap	215	No VT	(No)/60

*LV* left ventricle, *VT* ventricular tachycardia, *VF* ventricular fibrillation

### Surgical access

The pericardium was successfully accessed through a left lateral thoracotomy in all six patients. The severity of adhesions among patients varied remarkably: dense adhesions were found in the lateral to anterior portion of the LV in the three patients with previous cardiac surgery. In three patients with previous epicardial ablation, mild to moderate adhesions were found. Adhesions to the diaphragmatic portion of the pericardium and anterior wall were encountered in all patients, which contributed to the failure of percutaneous subxiphoid pericardial access. In patients with severe pericardial adhesions (patient #2, #4 and #5), blunt or sharp dissection of adhesions was performed as follows: the steerable sheath and mapping catheter could be advanced into pericardial space to separate the adhesion part, and then manipulated gently through the region of initial adhesions to reach the area of interest.

### Mapping and RF ablation

The clinical VT with a cycle length of 300–580 ms could be induced by programmed stimulation among five of the six patients. One patient had incessant VTs (cycle length: 300–480 ms) when he was transferred to the EP lab. A total of 15 different VTs were inducible in all 6 patients and the mean number of VTs per patient was 2.5 ± 1.9 (range 1–6).

**Fig. 2:**
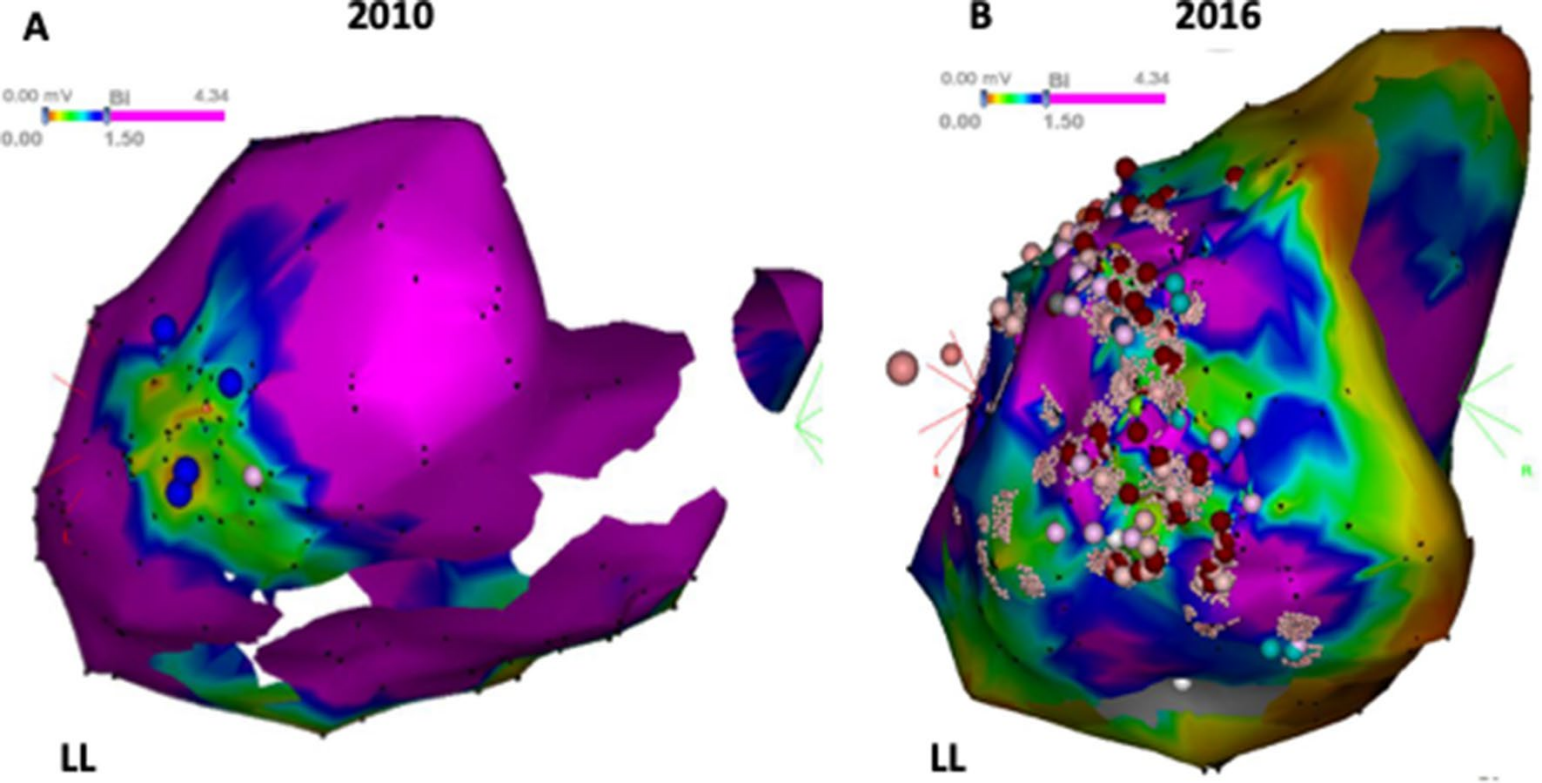
Epicardial voltage maps of patient #3. **a** Epicardial electroanatomical map from prior procedure in 2010. Sites where phrenic nerve captured by pacing from the map catheter (output: 10 mv 2 ms) were labeled by blue points. **b** Epicardial voltage map from a repeat procedure in 2016. Complete epicardial mapping and ablation were performed via the left lateral thoracotomy approach. Compared with map from 2010, more epicardial substrate with low voltage and fragmented potential was found in lateral-inferior of LV

**Fig. 3:**
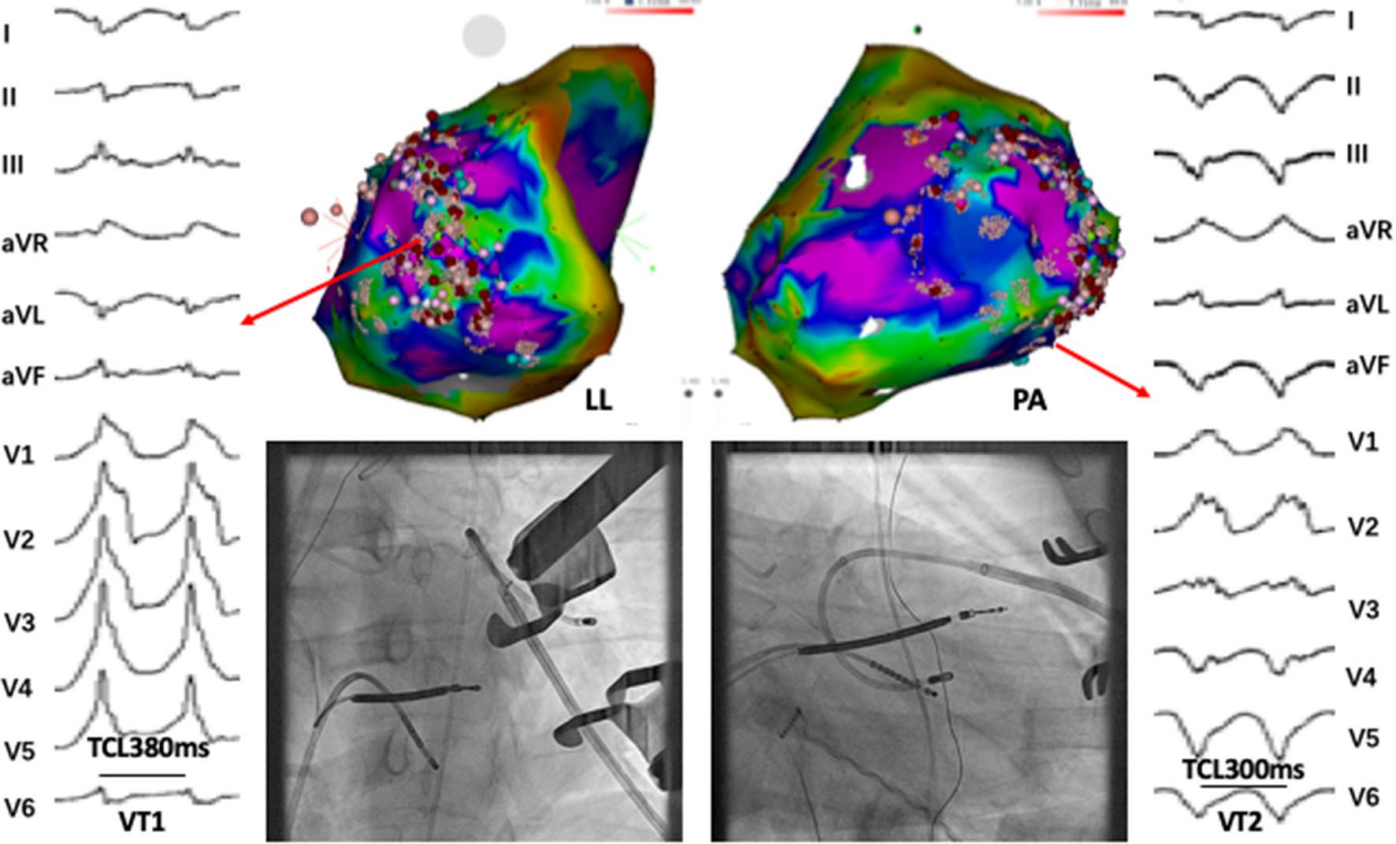
Clinical VTs and ablation target regions of patient #3. Ablation target of VT1 was located at left lateral basal of LV. VT2 was abolished after RF ablation delivered at inferior-basal of LV. *TCL* tachycardia cycle length

Complete epicardial mapping was acquired in two patients (patient #1, #3) with moderate adhesion which was encountered and lysed by manipulation of catheter and sheath. Patient #3 (DCM) underwent successful endocardial and epicardial VT ablation in 2010. The map demonstrated an endocardial substrate infero-basal of the right ventricle (RV) and an epicardial substrate within the infero-lateral region of the LV. However, the clinical VT was originated from the RV and was abolished by focal RFC ablation in the RV, epicardial ablation was not performed due to phrenic nerve capture and no evidence indicating any VTs originating from the LV (Fig. 2a). The patient had freedom of ventricular arrhythmias for 6 years after the initial ablation and was referred due to multiple ICD shocks, which presented with right bundle branch block morphology (Fig. 3). Since no abnormal substrate was found on the endocardial map, epicardial mapping was attempted; however, subxiphoid puncture failed due to pericardial adhesion (Fig. 1a). Pericardial space access was successfully obtained by surgical left lateral thoracotomy. Pericardial adhesions were visualized in the epicardial space. Epicardial mapping demonstrated a large substrate with a low-voltage area with fragmented and late potentials compared with the previous epicardial map (Fig. 2b). The clinical VTs were abolished by extensive RF ablation.

**Fig. 4:**
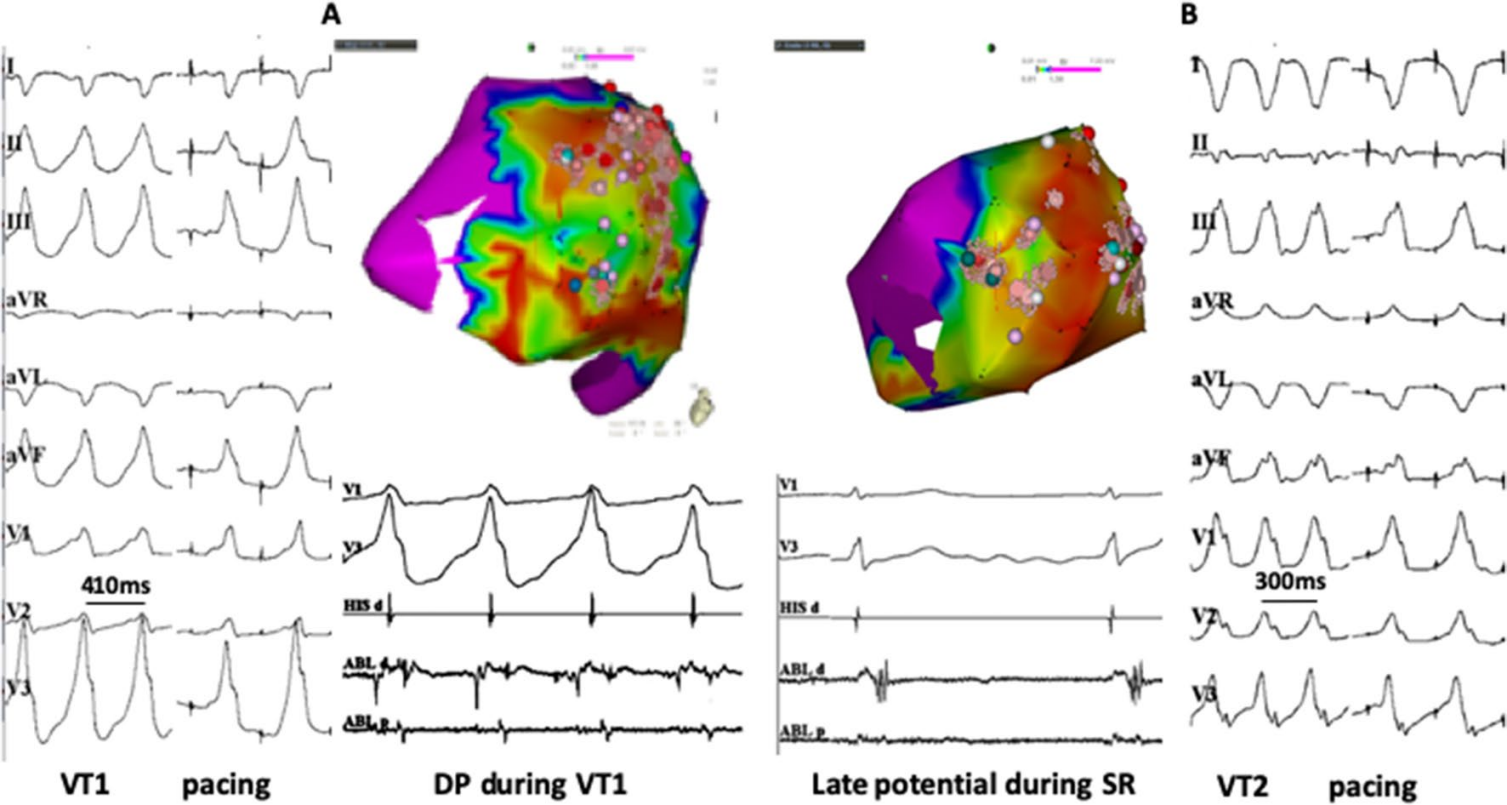
VTs and electro-anatomical map of patient #5. **a** Voltage map (LL view) of the epicardial surface exhibiting a large scar in the lateral to posterior wall of the LV, Pace mapping at superior lateral of scar produced QRS morphology similar to that of VT1. Stimulus-QRS interval during pace mapping at this site (92 ms) and diastolic potential (DP) during VT are consistent with slow conduction, possibly associated with VT reentry circuit. **b** Voltage map (LL view) of the LV endocardial surface, Pace mapping at lateral produced QRS morphology similar to that of VT2, late and fragmented potential during sinus rhythm are recorded at this site and stimulus-QRS interval during pace mapping is 80 ms. In this patient, the His catheter was located in RV apex. Precordial ECG leads V4 to V6 were displaced because of surgical incision

In the other four patients, mapping of the epicardial lateral basal and inferior wall of the LV was incomplete because catheter manipulation was limited by adhesions. The clinical VTs were abolished by epicardial ablation alone in two patients (#4 and #6). In patient #5, who had ICM and prior CABG surgery, additional LV endocardial mapping was performed after initial epicardial mapping and ablation due to a second VT remained inducible. The VT was identified and ablated successfully from the left lateral base of the endocardium (Fig. 4b). In patient #1, clinical VT was still induced after extensive ablation at the epicardial and endocardial LV. Bipolar ablation abolished the clinical VTs in the patient, in whom ST segment elevation of inferior leads was observed when delivering bipolar RF energy at the infero-basal LV, and complete occlusion of the proximal posterior descending artery was revealed by diagnostic coronary angiography. The vessel was subsequently dilated, and a drug-eluting stent was implanted. No clinical VT was induced even by aggressive programed stimulation up to three extra stimuli.

The total duration including the surgical pericardial access and ablation of the procedure was 250.8 ± 58.2 min (range 115–325 min) and the fluoroscopy time was 9.1 ± 7 min (range 3.1–16.9 min).

### Complications and follow-up

No periprocedural complications were observed in any patient except patient #1 with a total occlusion of the posterior descending artery during bipolar ablation. No severe consequences occurred after implanting a drug-eluting stent. All patients were discharged after a mean of 10.2 ± (7–15) days. During a median follow-up of 203 days (interquartile range 75, 900 days), no patients died. No VT recurred in 5/6 patients (83%). In patient #1 only one VT episode was documented in the ICD interrogation and was terminated with an anti-tachycardia pacing 23 months after the ablation procedure. The patient had no further episodes after oral administration of amiodarone 200 mg/day.

## Discussion

To our knowledge, this is the first case series describing the feasibility of the surgical left lateral thoracotomy approach for the treatment of scar-related VT located in the infero-lateral LV in patients with inaccessible pericardial access.

Access to the pericardial space could be successfully achieved via the surgical left lateral thoracotomy in all patients who had pericardial adhesions due to prior epicardial catheter ablation or cardiac surgery. This approach seems to be safe, feasible, and effective.

In patients with DCM and hypertrophic cardiomyopathy (HCM) who have a high probability of an epicardial substrate, epicardial ablation may be considered [6, 13, 14], especially for the patients with previous unsuccessful endocardial ablation procedures. However, the percutaneous subxiphoid pericardial approach is challenging in patients with a history of open-heart surgery or prior epicardial procedures because of the possibility of pericardial adhesions [8]. There are limited reports with repeat percutaneous pericardial access for ablation of recurrent VT despite the presence of significant adhesions from previous procedures [15, 16]. However, adhesions may prevent access or limit mapping, and risk of multiple RV punctures or RV pseudoaneurysm is highly increased [8, 17]. A hybrid procedure involving a surgical access may often be required under certain conditions [18]. In our study, at least one unsuccessful percutaneous subxiphoid pericardial approach was attempted before the surgical approach. Presence of pericardial adhesions in the inferior wall was revealed during the surgical access, and it is the most likely reason for an unsuccessful percutaneous subxiphoid pericardial access.

Several different hybrid surgical approaches have been reported so far. The subxiphoid surgical access to amend catheter-based epicardial ablation was first described by Soejima et al. in a case series of six patients with pericardial adhesions [19]. In the Moskowitz’s study, both a subxiphoid and a limited anterior thoracotomy approach were described [20]. Bradfield and colleagues described their experience with a sub-xiphoidal (*n =* 22) and anterolateral (*n =* 18) surgical epicardial access and the influence of the surgical approach and compared outcomes with the percutaneous epicardial access [21]. However, no lateral thoracotomy was performed in these patients [21]. Surgical incision positioning is critical when surgery access is required due to the differences in exposure area among different access approaches. ECG interpretation of the clinical VT, scar location, the pericardial adhesions and specific circumstances also need to be considered. It is reasonable for the patients to perform a subxiphoid surgical access because the majority of included patients in the studies mentioned above had an inferior or inferolateral scar and most of the target areas were located in those regions [20]. For the ICM patients with prior CABG, the pericardial adhesions are anticipated to be more severe in the anterior and lateral wall than in the inferior wall of the LV. In our series, the LV basal lateral region was part of the epicardial area of interest suggested by previous failed endocardial ablations or 12-lead ECG of VT. In most of these patients, a subxiphoid approach is difficult to access into this region, and a left lateral approach could provide more reliable exposure of the lateral basal and inferior region of the LV. In addition, the left phrenic nerve is usually in the proximity of the left atrial appendage and the basal inferolateral LV epicardium [22]. Therefore, a target region in close proximity to the phrenic nerve may prevent a successful epicardial ablation [23]. Compared to the subxiphoid approach, when ablation was performed close to the phrenic nerve, the parietal epicardium can be mechanically displaced from the mapping catheter tip during RF ablation to avoid phrenic never injury by the surgical lateral approach. A direct visualization of coronary arteries via a surgical approach potentially decreases the risk of artery injury. In the six patients with evidence of subepicardial substrate, 15 VTs were induced before ablation. After extensive epicardial ablation, VTs were still induced in patient #1 and #5; in patient #5, VT was eliminated with additional endocardial ablation, and VTs were abolished by bipolar ablation in patient #1. The data strongly suggest that in patients who had an indication for an epicardial substrate ablation, mechanisms of ventricular arrhythmia are complex and transmural substrates may be present.

### Safety of lateral surgical thoracotomy

Utilizing a surgical left lateral thoracotomy approach, the epicardial mapping and ablation could be achieved in all patients. However, a complete LV map was only achieved in two out of six patients, which indicates that our approach can be used only in patients with VT originating from the infero-lateral region of the LV. However, compared with the subxiphoid epicardial approach which may have potential risk of diaphragm injury, hepatic or abdominal bleeding, no surgery-related complications were observed in our study. Therefore, this approach should be considered in patients with suspected VT located at lateral basal and inferior regions of the LV and pericardial adhesions due to previous cardiac surgery and catheter ablation.

On the other hand, an ablation-related injury of the coronary artery was still found in one patient with bipolar ablation although the distance between coronary artery and target site was carefully assessed by coronary artery angiography and surgical visualization of the coronary arteries. This phenomenon may be explained by the larger lesion created by bipolar ablation or slight catheter movements in the epicardial space. It should be taken into account to avoid such complications in clinical practice.

A potential disadvantage of the left lateral thoracotomy might be as a relatively small operation window with limited direct visualization and difficulty in catheter stabilization. However, larger case series and trials are necessary to evaluate these issues.

## Conclusions

For selected patients with inaccessible pericardial access due to pericardial adhesions, the surgical left lateral thoracotomy approach is feasible, and can allow successful epicardial ablation procedures.

### Clinical competencies—patient care and procedural skills

A surgical left lateral thoracotomy for catheter ablation of scar-related left ventricular tachycardia is effective, feasible, and safe in patients with inaccessible pericardial access due to pericardial adhesions after previous cardiac surgery or epicardial ablation procedures.

### Translational outlook

Scar-related left ventricular tachycardia located at the left ventricular infero-lateral epicardial region can be effectively abolished via a surgical left lateral thoracotomy even under incomplete epicardial mapping in patients with failed percutaneous subxiphoid pericardial access. Although we are presenting only a limited number of cases, the data might (−) influence other physicians to perform this procedure in their patient population.

## Abbreviations

CABG: Coronary artery bypass graft
DCM: Dilated cardiomyopathy
ICM: Ischemic cardiomyopathy
LV: Left ventricle
LVEF: Left ventricular ejection fraction
RF: Radiofrequency
RV: Right ventricle
VT: Ventricular tachycardia
